# Low-level laser therapy in neurosensory recovery – case series in dental and maxillofacial rehabilitation

**DOI:** 10.1080/07853890.2021.1897361

**Published:** 2021-09-28

**Authors:** T. Nunes, S. Alves, M. Pimenta, S. Rocha, C. Caetano, A. Corte-Real

**Affiliations:** a Fifth-year student of Integrated Master in Dentistry, Faculty of Medicine, University of Coimbra, Portugal; b Faculty of Medicine, University of Coimbra, Coimbra, Portugal; c Forensic Dentistry Laboratory, Faculty of Medicine, University of Coimbra, Coimbra, Portugal

## Abstract

**Introduction:**

Neurosensory disorders may be considered as a complication from surgical procedures, such as dental implants and mandibular osteotomy [1–3]. In these cases, the orofacial damage can involve general and professional patient impairments. Low-level laser therapy (LLLT) has been reported to be effective in reducing neurosensory recovery time, while promoting nerve regeneration [4,5]. This study aimed to analyse the LLLT impact on postsurgical neurosensory recovery, namely for dental and maxillofacial surgery patients.

**Materials and methods:**

Patients previously submitted to dental and/or maxilofacial rehabilitation at Centro Hospitalar Universitário de Coimbra/Faculty of Medicine, University of Coimbra, were selected. Anamnesis, examination and neurosensory evaluation were performed to determine the presence and location of neurosensory disorder. The elected orofacial area was irradiated using a continuous wave diode laser at 660 nm (SIROLaser Blue; Sirona, Bensheim, Germany) in two sessions per week, until satisfactory results were achieved. The measure of health-related quality of life was performed by EQ-5D-5L questionnaire, before and after the LLLT treatment. This study was approved by the ethics committee of Faculty of Medicine of the University of Coimbra and the informed consent document was performed.

**Results:**

Two patients (both Female, Age 22) were selected. Treatment lasted approximately 1 months in both cases. The mean score before and after treatment (0–100 scale), of the EQ-5D-5L questionnaire was 70.0–95.5.The recovery of the neurosensory disorder allowed the quality improvement in all 5 dimensions: mobility, self-care, daily activities, pain/discomfort and anxiety/depression. No reactions or side-effects were reported.

**Discussion and conclusions:**

5 dimensions of health quality were analysed and the severity level degrees were related with the corporal/patient impairment. The results supported the use of LLLT as an effective treatment option by accelerating the recovery of postsurgical neurosensory disturbances, improving the patient’s health.
